# A Two-stage Peak Alignment Algorithm for Two-Dimensional Gas Chromatography Time-of-Flight Mass Spectrometry-Based Metabolomics

**DOI:** 10.5936/csbj.201304002

**Published:** 2013-06-19

**Authors:** Bing Wang

**Affiliations:** a The Advanced Research Institute of Intelligent Sensing Network, Tongji University, shanghai, 201804, China; b The Key Laboratory of Embedded System and Service Computing, Ministry of Education, Tongji University, Shanghai 201804, China; c School of Electronics and Information Engineering, Tongji University, Shanghai 201804, China

**Keywords:** GC×GC/TOF-MS, peak alignment, retention time, spectrum similarity, metabolomics

## Abstract

Comprehensive two-dimensional gas chromatography coupled with time-of-flight mass spectrometry (GC×GC/TOF-MS) has been applied to metabolomics analyses recently. However, retention time shifts in the two-dimensional gas chromatography will introduce difficulty to compare compound profiles obtained from multiple samples. In this work, a novel two-stage peak alignment algorithm has been developed for data analysis of GC×GC/TOF-MS. In the first stage, our algorithm detects and merges multiple peak entries of the same metabolite into one peak entry. After a z-score transformation of metabolite retention times, landmark peaks will be selected from all samples based on both two-dimensional retention times and mass spectrum similarity of fragment ions measured by Pearson's correlation coefficient. In the second stage, the original two-dimensional retention time shift will be corrected using a local linear fitting method. A progressive retention time map searching method is used to align peaks in all samples together based on the parameters optimized in the first stage. Our algorithm can avoid defining a threshold of retention time window and spectrum similarity, which is very difficult for the users since the experimental condition is always changed in different experimental runs, even for the repeat experiments. The experimental results show that our algorithm can work well in peak alignment from real biological samples, which is very important for the further analysis.

## 1. Introduction

An emerging technology, comprehensive two-dimensional gas chromatography coupled with time-of-flight mass spectrometry (GC×GC/TOF-MS), brings much increased signal-to-noise ratio, dynamic range, chemical selectivity, and sensitivity to metabolomics analyses [1–4]. This approach uses a multidimensional separation technique, where a short column after the main analytical column, to separate as many compounds as possible [5, 6]. The orthogonal setup of two columns in separation part makes GC×GC/TOF-MS platform get an order-of-magnitude increase in separation capacity, which is very important for the analysis of many complex samples.

After analyzing these samples using GC×GC/TOF-MS, it is necessary to recognize molecular features of the same compound occurring in different samples from each of the raw instrument data [7, 8]. Ideally, the same compound should have the identical retention times in the two-dimensional GC if the instrument configuration is the same. However, retention times always shift in both GC dimensions as a result of several, sometimes uncontrollable factors such as temperature and pressure fluctuations, matrix effects on samples, and stationary phase degradation. Retention time shifts introduce difficulty to compare compound profiles obtained from multiple samples. Therefore, aligning the instrumental signals which generated from same compounds in different samples, i.e., peak alignment, have to consider the retention time variation [9].

Currently, four studies addressed alignment issue for the two-dimensional GC separations using the raw instrument data as input material. Fraga et al. developed a rank-based algorithm using the generalized rank annihilation method (GRAM) to correct retention time variations in the two- dimensional GC [10, 11]. Mispelaar et al. developed a correlation-optimized shifting-based algorithm to align a local region of a GC×GC chromatogram [12]. These two methods can only be used to align small regions of interest in the two-dimensional GC data set. To correct the entire chromatogram in both GC dimensions, Pierce et al. proposed an indexing scheme together with a piecewise retention time alignment algorithm [13]. Zhang et al. developed a two dimensional correlation optimized warping (2-D COW) method by extending the correlation optimized warping method from 1-D to 2-D [14, 15]. However, these methods align the GC×GC/TOF-MS data based on two-dimensional retention times alone, even though the signature feature of a metabolite, i.e., mass spectrum of fragment ions, is readily available in the raw instrument data. Aligning metabolite peaks solely based on the two dimensional retention times may introduce a high rate of false alignment because some metabolites with similar chemical functional groups have similar retention times in both GC dimensions. For this reason, two peak alignment methods, MSort [16] and DISCO [17], were developed. In these two methods, the raw instrument data are first subjected for spectrum deconvolution to generate a list of metabolite peaks for each sample, of which each metabolite peak is characterized by multiple molecular features including retention times in the two-dimensional GC, peak area, fragment spectrum, and other associated features. Both of MSort and DISCO employ two dimensional retention times and the mass spectrum of compound fragment ions for peak alignment. MSort was designed to align homogeneous data, while DISCO can align homogeneous and heterogeneous data. These two methods greatly reduced the rate of false alignment compared to existing alignment approaches. However, MSort and DISCO softwares use a user-defined retention time window with a fixed size in the two retention time dimensions to filter the peak candidates first, then the peak pair with the highest similarity will be choose as corresponding peaks in the different experiments if its value is bigger than a user-defined threshold. The separated application of retention time distance and spectrum similarity increase false alignment since there is no a golden criteria to select the distance window and the spectrum similarity threshold.

To overcome the limitations of current alignment algorithms, this paper reports a novel alignment algorithm for GC×GC/TOF-MS which can consider the distance and spectrum similarity together and automatically set up the threshold values. After the peak lists are generated from instrumental software, the present algorithm is implemented using a two-stage strategy. In the first stage, landmark peaks, a set of compound peaks present in each biological sample, are selected from the entire original peak lists using a mixture similarity comprised of the Euclidean distances of two-dimensional retention times and mass spectrum similarity of two corresponding peaks. In the second stage, retention time shifts are corrected using a local partial linear fitting method to handle non-linear retention time distortion, and the compound peaks of all samples then are aligned using a progressive retention time map searching method.

## 2. Methods

**Figure 1:**
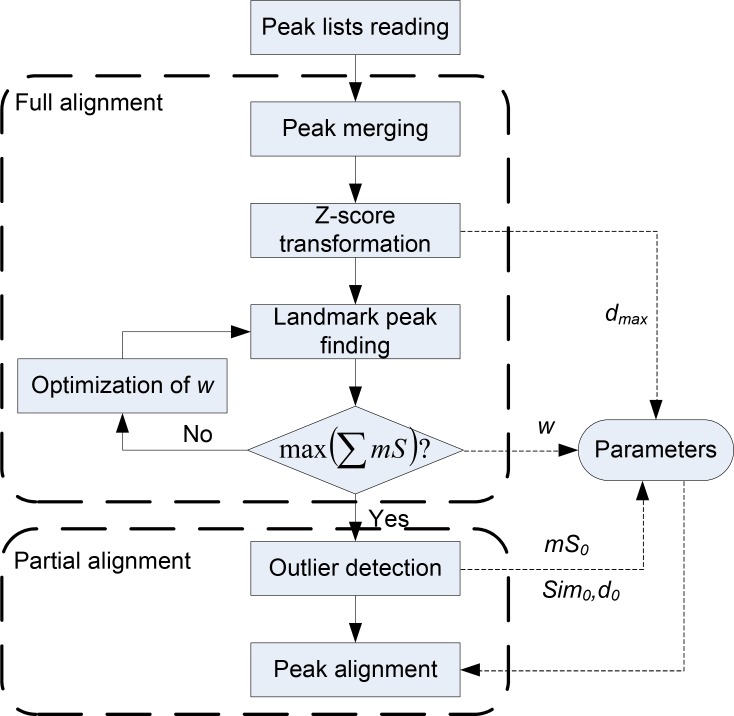
Workflow of the two-stage peak alignment algorithm.

For each experimental run, ChromaTOF will generate a peak list analyzed by instrumental software under predefined parameters. For all of the peak lists, our algorithm will align them using a two-stage peak alignment algorithm: stage one is full alignment which will find the peaks present in all lists and align them together; stage two is partial alignment which will align all remained peaks from stage one based on the results of full alignment. The workflow can be described as Figure 1.

In our method, a mixture similarity measurement is adopted to judge two peaks from different peak list are from the same compound or not. This mixture similarity *mS* can be calculated as follows:

1 $$\text{mS}=w\times (1-{\left(d/d_{\max}\right)}^{2})+(1-w)\times Sim$$

where W is contribution factor, *d* is the Euclidean distance of two peaks in two-dimensional retention time space, *d_max_* is the maximum distance for all peaks within all samples, and sim is Pearson's correlation value of spectra of two peaks.

The alignment algorithm is implemented using a two-stage strategy: full alignment and partial alignment. In the full alignment, the peaks presented in all peak lists will be found and aligned together. Meanwhile, the parameter *w* and *d_max_* in the formula (1) can be obtained for the partial alignment.

### 2.1 Full alignment

#### 2.1.1 Peak merging

Assuming there is a set of peak lists:*S*(*s*_1^,^_ *s*_2_, L, *s*_*N*_), our algorithm will merge the peaks which come from the same compound in each peak list. Ideally, all instrument signals generated by one type of metabolite should be reported as a single peak in the output file of ChromaTOF software, i.e., one peak entry in the metabolite peak list. However, multiple peak entries of the same metabolite can be reported due to the abnormal metabolite peak shape and/or the high sensitivity of the peak detection algorithm. Therefore, the multiple entries should be merged before peak alignment.

1. Initialize a user-defined retention time window RT1w, RT2w, and a user-defined mass spectrum similarity *R*_0_, set *i=*1*,j=*1;
2. For the *i* ^*th*^peak *P*_*i*_^(*rt*1i,*rt*2i)^ in the *j* ^*th*^ peak list *s*_*j*_, if the *i* ^*th*^ peak is the last peak in the peak list *S*_*j*_, set *i=*1*,j=j+*1 when *j<N*, or stop when *j=N*, otherwise our algorithm will store this peak into a peak set $P_{candiate}^{final}$ and extract all peaks within (*rt*1_i_,*rt*1+*RT*1w) as $p_{candiate}^{rt1}$

3. If $p_{candiate}^{rt1}$ is not empty, extracts all peaks within (*rt*2_i_,*rt*2_i_+*RT*2w)as $p_{candiate}^{rt2}$ within $p_{candiate}^{rt1}$ , else set *i=i*+1 go to step 2);
4. If $p_{candiate}^{rt2}$ is not empty, the spectrum similarity between *P*_*i*_ and all the peaks in $p_{candiate}^{rt2}$ will be calculated. If there are some peaks have similarity values which are bigger *R*_0_, those peaks can be seen as multiple entries candidate *P*_*candiate*_ of *P*_*i*_, else set *i=i*+1 go to step 2);
5. If *P*_*candiate*_ is not empty, store *P*_*candiate*_ into $P_{candiate}^{final}$ , let *rt*1_i_,*rt*1+*RT*1w and go to step 2), else if the number of peaks in $P_{candiate}^{final}$ is bigger than 1, our algorithm will extract all the peaks in $P_{candiate}^{final}$ from the peak list *S*_*j*_, then merge them together as a new peak within *S*_*j*_ and set its peak area and retention time as:
2 $$A_{p_{n}}=\sum_{i=1}^{k}A_{p_{i}}$$
3 $$RT_{p_{n}}=\frac{1}{A_{p_{n}}}\sum_{i-1}^{k}A_{p_{i}}*RT_{p_{i}}$$
  where $A_{p_{n}}$ denotes peak area of the representative peak, $A_{p_{i}}$ is peak area of the ith multiple entry to be merged, *k* is the index number of multiple peak entries to be merged, $RT_{p_{n}}$ denotes retention time of the representative peak, $RT_{p_{i}}$ is the retention time of the ith multiple entry to be merged. Here, the retention time is either the first dimension retention time or the second dimension retention time.
6. Set *i=i+1* and go to step 2);

#### 2.1.2 Z-score transformation

For typical GC×GC/TOF-MS configuration, the second GC column is much shorter than the first column, which will result in the second retention time is smaller than the first retention time obviously. To balance the contribution of the two dimensional retention times, the retention time values in both the first and the second dimension GC are therefore transformed into z-scores as following:

4 $$RT_{1z}=\frac{RT_{1}-RT_{1\mu }}{RT_{1\sigma }},RT_{2z}=\frac{RT_{2}-RT_{2\mu }}{RT_{2\sigma }}$$

where *RT*_1z_ is the z-score value after transformation, *RT*_1_ is the original value of the first dimension retention time, *RT*_1µ_ is the mean value of the original first dimension retention times within a peak list, *RT*_1σ_ is the standard deviation of the original first dimension retention times. Accordingly, the symbols in the second dimension retention time have similar meanings. For all the peaks in all peak lists, the pairwise distances will be calculated and the maximum value is chosen as *d*_*max*_.

#### 2.1.3 Landmark peak finding

The purpose of full alignment is to find a list of landmark peaks which present in all peak lists. Therefore, one of peak lists has been selected randomly from sample set *S*(*s*_1^,^_ *s*_2_, …, *s*_*N*_) as reference sample *S*_*R*_, and the remain are seen as target sample set $\bar{S}=\left\{S_{i}\right\}\left(i=1,2\ldots ,N-1\right)$ . Then landmark peaks can be found as follows:

1. Selecting a sample randomly as the target sample *S*_*T*_ from the set of remaining samples $\bar{S}$ , then $\bar{S}=\left\{S_{i}\right\}\left(i=1,2\ldots ,N-1\right)$ .
2. For each peak *P*_*r*_in the reference sample *S*_*R*_, our algorithm will extract the peaks with same name from the target sample *S*_*T*_ as a candidate peak set $P_{candidate}^{sameName}$ , and choose the one with the minimum distance from *P_r_* in z-score transformation space of retention time as the corresponding peak *P*_*t*_of *P*_*r*_ in *S*_*r*_.
3. Storing the peak pair (*P*_*t*_, *P*_*r*_) into a set of landmark peaks *P* ^*lp*^.
4. Updating the reference sample *S*_*R*_ using the peaks which has found the corresponding peak, and if the number of sample in $\bar{S}$ is not one, go to step 1), else stop

After landmark peaks set *P^lp^* has been found, we can get an optimized value of *w* from a candidate value set (0.05,0.1,0.2,0.3,0.4,0.5,0.6,0.7,0.8,0.9,0.95). For each sample pair (*S*_*R*_, $S_{T}^{i}$ )where *S*_*R*_ is the reference sample and $s_{T}^{i}(i=1,2,\cdots ,N-1)$ from the remained samples, our algorithm calculate the mixture similarity *mSim* of the each landmark peak using the formula (1), and get a summary value *mS* for each sample pair. And choose the *w* value with the largest summary value of *mS* as the optimized value for each sample pair.

### 2.2 Partial alignment

The partial alignment is to align the remained peaks which are not the landmark peaks selected the full alignment. After full alignment, we already get the value of *d*_*max*_ and *w* in formula (1) and a set of landmark peaks which present in all the samples. However the remained peaks also should be aligned together even they present in part of samples. Our algorithm will use an outlier detection method to decide the threshold of distance and similarity automatically, and implement partial alignment based on those threshold values.

#### 2.2.1 Outlier detection

Our algorithm considers the landmark peaks as true peak alignment, therefore the information from the landmark peaks can be used to help the partial alignment because the remained peaks are detected by the same experimental conditions and the same data processing procedure. Here we select the maximum peak distance *d*_0_, the minimum similarity *sim*_0_ and the minimum mixture similarity *mS*_0_ as threshold to judge the remained peaks from different sample come from same compound or not.

However, because the complexity of sample source and some inevitable variation from experiment, the data points will be further away from the mean value of the above three parameters than what is deemed reasonable. The simple selection of the extreme value of parameters as the threshold will cause faulty alignment of peaks. Therefore, our algorithm remove the outliers using Grubbs’ test method from the above three parameters, and set the threshold values *d*_0_,*sim*_0_ and *mS*_0_after outlier removal.

#### 2.2.2 Peak alignment

Our algorithm aligns the remained peaks in a pair wise way, i.e.(*S*_*R*_, $S_{T}^{i}$ ) where *S*_*R*_ is the reference sample and $s_{T}^{i}(i=1,2,\cdots ,N-1)$ from the remained samples. For each sample pair(*S*_*R*_, $S_{T}^{i}$ ), the remained peaks are aligned as follows:

1. For each peak *P*_*i*_ in *S*_*R*_, the peak *P*_*i*_ in $S_{T}^{i}$ , if they satisfy the criteria that *d*(*p*_*i*_, *p*_*j*_)<*d*_0_ and *Sim*(*P*_*i*_, *P*_*j*_)>*Sim*_0_, the peak pair (*p*_*i*_ *, p*_*j*_) will be selected as a candidate peak pair and store into a candidate set $P_{candidate}^{i}$ .
2. Within $P_{candidate}^{i}$ , all peak pairs have the same name which assigned by ChormaTOF software will be extracted as a subgroup $P_{candidate}^{sameName}$ , and assign the remained peak pairs as another subgroup ${\bar{P}}_{candidate}^{sameName}$ .
3. For each compound name, we account the times present within $P_{candidate}^{sameName}$ , if it just presents in one peak pair, this peak pair will be seen as the same compound and transfer this pair into aligned peak pair set $P_{aligned}^{i}$ ; if it presents in more than one peak pair, the peak pair with the largest *mS* value will be transferred into $P_{aligned}^{i}$ , and remove all pairs in $P_{candidate}^{sameName}$ consists of either peak of this peak pair. Repeat this step until $P_{candidate}^{sameName}$ is empty.
4. Removing all peak pairs in ${\bar{P}}_{candidate}^{sameName}$ with the same peak within $P_{aligned}^{i}$ .
5. If ${\bar{P}}_{candidate}^{sameName}$ is not empty, our algorithm will transfer the peak pair with the largest *mS* value from ${\bar{P}}_{candidate}^{sameName}$ into $P_{aligned}^{i}$ , and remove all pairs in $P_{aligned}^{i}$ consists of either peak of this peak pair. Repeat this step until ${\bar{P}}_{candidate}^{sameName}$ is empty.
6. The above steps 1)-5) will be repeated until all sample pair (*S_R_*, $S_{T}^{i}$ ) (*i* =1,2,⋯, *N* −1) are aligned and all $P_{aligned}^{i}$ are combined into partial alignment result *P*_*aligned*_.
7. After partial alignment, our align algorithm will be finished with combination of full alignment result *P* ^*lp*^and partial alignment result *P*_*aligned*_.

## 3. Results and discussion

### 3.1 experimental data

A mixture of 76 compounds (8270 MegaMix, Restek Corp., Bellefonte, PA) and C7-C40 saturated n-alkanes (Sigma-Aldrich Corp., St. Louis, MO) were spiked with a deuterated six component semi-volatiles internal standard (ISTD) mixture (Restek Corp., Bellefonte, PA) at a concentration of 2.5 µg/mL prior to GC×GC/TOF-MS analysis.

The performance of our algorithm was tested by aligning three datasets, which are replicate analyses of the same sample using an identical two dimensional GC configuration with different column temperature gradients, i.e., 5 °C /min, 7 °C/min, and 10 °C /min, respectively. To evaluate the performance of our proposed algorithm more objectively, the experiments ramped at different temperature gradients have been repeated different times, i.e., 10 replicate analyses for 5 °C /min, 3 replicate analyses for 7 °C/min, and 4 replicate analyses for 10 °C/min. The design of the different repeat times of different experimental conditions will help us to evaluate how robustness our algorithm is. The experiments can be found in our previous work in more details [17].

### 3.2 performance measurements

Here the algorithm performance is evaluated by three measurements, i.e., true positive rate (TPR), positive predictive value (PPV), and F1 score of the peak alignment. For the sample set *S(S*_*1*_ *,S*_*2*_ *,...S*_*N*_ *)*, if a compound presents in all N samples, it will be called as a positive peak pair. After peak alignment, if the number of positive peak pairs is *N*_*P*_and the number of matched peak pairs is *N*_*m*_, then the values of TPR, PPV and F1 score can be calculated as follows:

5 $$\begin{array}{c} \text{TPR}=\text{TP}/(\text{TP}+\text{FN})\\ \text{PPV}=\text{TP}/(\text{TP}+\text{FP})\\ F1=2\cdot \text{TPR}\cdot \text{PPV}/(\text{TPR}+\text{PPV}) \end{array}$$

where TP is the number of positive peak pairs that were aligned as positive (true positive), FP is the number of negative peak pairs that were aligned as positive (false positive) and is *N*_*m*_-TP, FN is the number of positive peak pairs that were not aligned (false negative) and is *N*_*p*_-TP. TPR is also called recall and PPV precision and F1 score is their harmonic mean.

### 3.3 Parameters finding

**Figure 2:**
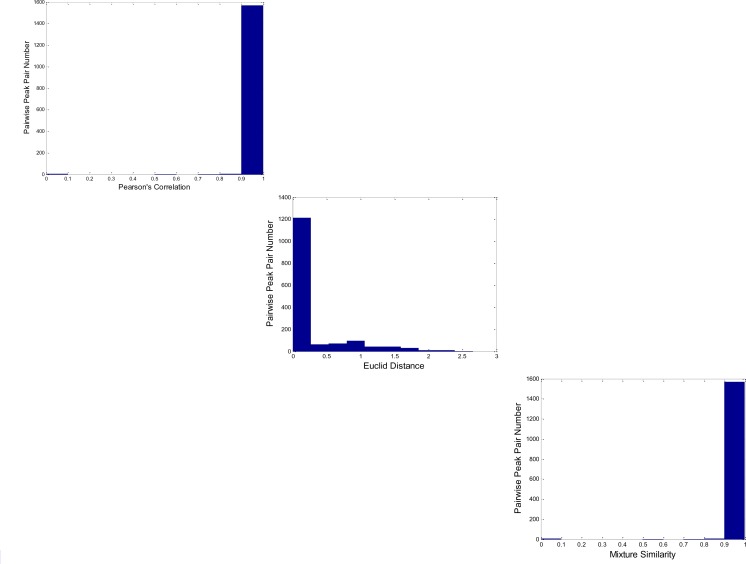
The distribution of Pearson's correlation, Euclid distance and mixture similarity of peak pairs.

One of advantages of our algorithm proposed here is there are no predefined parameters when the peak alignment is implemented. The method can automatically decide the threshold of Pearson's correlation of two spectra, the distance window in z-score transformation retention time space and the mixture similarity value. However, the complexity of samples and some inevitable variations of experiments may make the distributions of these parameters not normal. Our algorithm decides the thresholds based on the landmark peaks, which are presented all the samples. Except the information of retention time and spectrum similarity, our algorithm also uses the peak name assigned by the ChromaTOF software. After the full alignment, the distribution of parameters: Eculid distance, spectrum similarity *mS*, and mixture similarity *mSim* can be found in Figure 2. It can be seen that the each distribution of three parameters has an apparent tail. Therefore, the simple selection of maximum or minimum of these parameters may cause some inaccurate setting. Our algorithm uses an outlier detection method to remove the observations which are distant from the rest of the parameters, which makes the selections more reasonable.

### 3.4 Alignment performance

**Figure 3:**
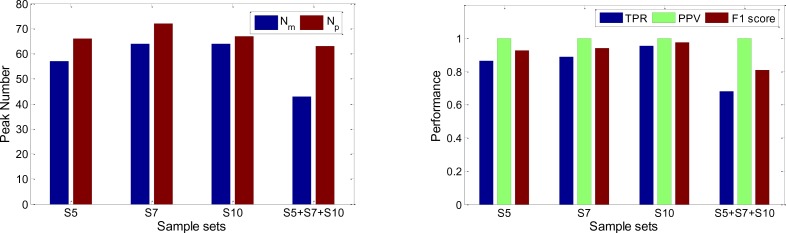
The overall performance of peak alignment.

Our algorithm is tested using the experimental data which are generated under different temperature gradient setup, i.e., 5 °C/min, 7 °C/min, and 10 °C/min. Therefore, 17 peak lists can be obtained from ChromaTOF where each peak list means one sample analyzed by GC×GC/TOF-MS, and they can be denoted as $S5(s_{1}^{5{}^{\circ}C},s_{2}^{5{}^{\circ}C},\text{L},s_{10}^{5{}^{\circ}C})$ , $S7(s_{1}^{7{}^{\circ}C},s_{2}^{7{}^{\circ}C},s_{3}^{7{}^{\circ}C})$ , and $S10(s_{1}^{10{}^{\circ}C},s_{2}^{10{}^{\circ}C},s_{3}^{10{}^{\circ}C},s_{4}^{10{}^{\circ}C})$ for the different temperature gradients respectively. After peak alignment, the overall performance can be found in Figure 3.

Obviously, the performance will be changed along with the sample size because the more samples will be aligned, the fewer peaks will present in all samples, and the fewer peaks will be aligned together. The number of positive peak pairs *N*_*p*_ is 72 for the 7, 67 for 10, 66 for 5 and 63 for all degree gradients experiments. The number of matched peak pairs *N*_*m*_ is 64, 64, 57 and 43 for 7, 10, 5 and all degree gradients experiments.

For Figure 3, we can also found that the PPV values our algorithm achieved in all different data sets are 1.0, which means false positive is zero based on the above definition of PPV. Our alignment algorithm can make sure the peaks what we aligned together are come from the same compound, therefore it can solve the false positive problem which is the most limitation of current peak alignment methods.

## Conclusions

This paper proposed a new two-stage peak alignment algorithm, which is consist of full alignment and partial alignment, to align the GC×GC/TOF-MS data. The present algorithm uses a set of peak lists which is output of the instrument control software, ChromaTOF, as its input data. In the full alignment, multiple peak entries of the same compound were detected firstly and merged into one peak. A z-score transformation was applied to balance the contribution of the first and second dimensional retention times in the peak distance calculation. Landmark peaks then were found based on the information from two-dimensional retention times, the mass spectrum similarity and the assigned compound name by ChromaTOF. In the partial alignment, our algorithm can automatically set up the threshold of peak distance, mass spectrum similarity and mixture similarity value to recognize the same compound from different experiments. A progressive retention time map searching method then is used to align metabolite peaks in all samples together based on the landmark peaks found in the full alignment.

Our algorithm can avoid a user-defined threshold of retention time window in the first and second dimension, as well as a threshold of spectrum similarity, which is very difficult task for the users since the experimental condition is always changed in different experimental runs, even for the repeat experiments. This kind of design can avoid the problems caused by improper parameter selection and inconsistency among samples. Another advantage of this work is taking full advantage of the information generated by experimental instrumental software, i.e. the two dimensional GC retention times, fragment ion spectrum correlation and database information which implicated by the compound name assigned by ChromaTOF.

The performance of the present algorithm was tested by experimental data where the samples were analyzed under different experiment conditions. The results show that our algorithm can work well in peak alignment from real biological samples. As a critical step of data pre-processing, the alignment results achieved by our propose methods can be used effectively for further analysis such as pattern recognition and statistical significance testing.
